# Clinical Practice Guidelines for the Last Days of Life: A Systematic Review

**DOI:** 10.3390/jcm15124407

**Published:** 2026-06-06

**Authors:** María Jesús de la Ossa-Sendra, Virginia P. Aguiar-Leiva, Inmaculada López-Leiva, Rosa Cazorla-Gonzalez, Jose M. Lapeira-Cabello, José M. Morales-Asencio

**Affiliations:** 1 Cudeca Foundation, Avda. del Cosmos s/n, 29631 Benalmádena, Spain; mariajesusossa@cudeca.org (M.J.d.l.O.-S.); vaguiarleiva@gmail.com (V.P.A.-L.); rosacazorla@cudeca.org (R.C.-G.); josemanuellapeira@cudeca.org (J.M.L.-C.); 2 Department of Nursing, Faculty of Health Sciences, Universidad de Málaga, Calle Arquitecto Francisco Peñalosa 3, 29017 Malaga, Spain; mainma@uma.es; 3 Instituto de Investigación Biomédica de Málaga (IBIMA-Bionand), Calle Severo Ochoa 34, 29590 Malaga, Spain

**Keywords:** palliative care, hospice and palliative care nursing, hospice care, palliative medicine, practice guidelines, patient care, hospice patients, terminal care, end of life, last days of life

## Abstract

**Background/Objectives:** To identify and evaluate contemporary clinical practice guidelines for the care of adult patients in their last days of life, their families and caregivers. **Methods:** A systematic review was conducted following the Preferred Reporting Items for Systematic Review and Meta-Analyses (PRISMA) statement (PROSPERO: CRD42021258311). PubMed, TRIP Database, Cochrane Library, ProQuest, and CINAHL were searched for CPGs published between January 2016 and December 2025 in English or Spanish, supplemented by searches of seven palliative care organisation websites. Two independent reviewers screened records; the AGREE II instrument was applied by four evaluators to assess methodological quality. Recommendations from included guidelines were categorised into 12 inductively derived decision areas encompassing recognition of the dying phase, communication and decision-making, multidisciplinary care, symptom management, and grief and bereavement. A narrative synthesis was adopted due to heterogeneity in guideline structure and recommendation grading systems. **Results:** Of 1118 records identified, 20 were retrieved for full-text assessment. Sixteen met CPG criteria and were appraised with AGREE II, of which eight were finally included. Included guidelines originated from five countries (UK, Spain, USA, Canada, and Europe) and were published between 2018 and 2021. Overall AGREE II scores ranged from 71% to 100%, with the lowest domain scores consistently found in Applicability. Strong recommendations were identified across most guidelines for recognition of the LDS, communication, and interdisciplinary coordination; recommendations on symptom management were mixed. **Conclusions:** Findings may inform professionals and health system managers in identifying key LDS care recommendations. Gaps in social, cultural, and spiritual dimensions should guide future guideline development. Key limitations include heterogeneity in guideline methods and restriction to English and Spanish publications. No external funding was received.

## 1. Introduction

Palliative care is aimed at improving the quality of life of patients and their families who face situations of advanced and terminal illness. It aims to alleviate suffering, pain and any other problem in the physical, psychological and spiritual spheres that may arise in this period that will end with the death of the patient [1]. The last days of life situation (LDS) is defined in the palliative care setting as the period in which death is imminent [2]. The duration of this period varies among patients, but it is recognised that it can last from a few hours to 3–5 days. Signs evident in the patient come from the deterioration of all vital functions and can be established abruptly, slowly, or very slowly [3].

It is important to recognise the moment when patients in the terminal phase of their illness go into their last days of life, as this change involves a worsening and intensification of suffering in the physical, psychological, and spiritual spheres of both the patient and their relatives and caregivers [4].

End of life is a common and frequent term used in the clinical setting and in scientific research, although not always with comparable implications [5]. It has been extensively conceptualised by different authors and groups [6,7]. The terminology used to describe the final phase of life is notoriously inconsistent in both clinical and research contexts. Hui et al. conducted a systematic review identifying substantial heterogeneity in the definitions and scope of concepts such as ‘actively dying’, ‘end of life’, ‘terminally ill’, ‘terminal care’, and ‘transition of care’ [6].

In the context of this review, last days of life refers specifically to the period of active dying (typically spanning from a few hours to 3–5 days) which is distinct from the broader concept of ‘end-of-life care’, used to refer to care during the final months or years of life. This distinction is clinically relevant, as the recognition of the dying phase triggers specific care decisions that differ substantially from those applicable to earlier stages of advanced illness.

It is well reported that healthcare staff do not notice the transition to the last days or have an inaccurate prediction of how long patients will live [8]. Recognising and expressing this change can be a source of conflict between professionals and relatives or carers, as well as confusion for the patient. Feelings of abandonment, hopelessness, anger, and bewilderment have been described in both the patient and the relatives and caregivers, even in healthcare staff. Some of the most conflictive decisions such as the withdrawal of nutrition and hydration or the deprescription of drugs for curative purposes (in favour of others for palliative and comfort purposes) are always delicate points to discuss with the family and even within the healthcare team itself [9].

Moreover, inequality in palliative and end-of-life care has been demonstrated across multiple dimensions, including the place of death, timeliness of access to specialist palliative care, and the extent to which social, spiritual, and cultural needs are met. Older patients, those with lower educational attainment, those living alone, and those dying in hospital have been shown to be less likely to receive palliative care consultation in their last week of life. Among ethnic minority groups, additional barriers arise from limited health literacy, language differences, migratory experiences, distrust of healthcare services, and the insufficient integration of cultural and religious values into care. These inequities highlight the need for more comprehensive, person-centred, and culturally sensitive approaches to care in the last days of life [10,11,12,13].

Several initiatives have been developed in the last 20 years to improve the identification and coordination of care at the end of life. The International Collaborative for Best Care for the Dying Person (ICBCDP) is a group of leading thinkers, practitioners, and researchers from 24 countries, united by the common goal of improving care for dying people and their families. In 2013, ICBCDP developed the 10/40 model. It is derived from ten key elements of care necessary for achieving the best care for the dying patient and 40 goals of care that transcend international and cultural boundaries and apply irrespective of the place of care. Since 2013, the 10/40 model has evolved and has been adopted by 22 different member countries [14].

The End-of-Life Research Group (EOLRG) has developed and implemented the care plan CAREFuL—Care Programme for the Last Days of Life—in acute geriatric hospital wards in Flanders, Belgium. The CAREFuL care programme was created to improve the quality of dying in patients. It achieves this by raising awareness of the importance of improving end-of-life care, training staff in the delivery of good end-of-life care with the support of a multi-professional care guide and regularly discussing the delivered end-of-life care in staff meetings. The programme involved a care guide for the last days of life, supportive documentation for healthcare staff and family caregivers, and an implementation guide [15].

On the other hand, clinical practice guidelines (CPGs) that clearly define when and how to act when a patient is in the LDS may contribute to contextualising and guiding the actions of the healthcare team in charge of their care and help to reduce the uncertainty and variability that surrounds decision-making in the LDS. There are many CPGs available from various organisations and groups that provide recommendations for the LDS, but there is no review on the consistency and rigour of their development.

The choice to focus on CPGs, rather than clinical pathways or other types of interventions, is grounded in the evidence on the limitations of the latter. A Cochrane review dating back to 2016 that examined clinical pathway initiatives concluded that there is a lack of consistent evidence regarding the clinical, physical, psychological or emotional effectiveness of end-of-life care pathways [16]. CPGs, by contrast, systematically synthesise the available evidence and provide explicitly graded recommendations, offering a more rigorous and reproducible framework for guiding clinical decision-making during the last days of life. Despite their potential value, no systematic review had previously evaluated the consistency and methodological quality of CPGs specifically addressing this phase of care.

Stakeholders involved in care during the LDS can benefit from an evaluation of the quality of the available CPGs on the LDS, the level of evidence underpinning the recommendations, and the identification of potential disparities in treatment recommendations.

## 2. Materials and Methods

The research question was structured according to the PICO framework (Population, Intervention, Comparison, and Outcome) (Table 1).

The aim of the study was to identify and evaluate contemporary CPGs (2016–2025) in the field of end-of-life care for adult patients in the LDS, their families, and caregivers.

The design of this study was a systematic review in accordance with the Preferred Reporting Items for Systematic Review and Meta-Analyses (PRISMA) statement [17]. The review protocol was registered in PROSPERO in July 2020 (CRD42021258311), Table S1: Recommendation in selected Guidelines.

Clinical practice guidelines were included if they were defined as ‘statements that include recommendations intended to optimize patient care that are informed by a systematic review of evidence and an assessment of the benefits and harms of alternative care options’ [18].

Clinical practice guidelines without geographic restrictions, addressing exclusively or partially the care of adult patients in the LDS were considered for inclusion. LDSs provided in highly specialised settings such as an Intensive Care Unit (ICU) were excluded. Systematic reviews and studies on the economic evaluation of end-of-life interventions were not considered for inclusion but were reviewed for potentially relevant papers.

A search of the PROSPERO database identified that no recorded systematic reviews on guidelines about this topic had been previously conducted or were currently in progress. For the localization of CPGs, search strategies were developed in the following sources: PubMed, TRIP database, Cochrane Library, ProQuest, Cumulative Index of Nursing and Allied Health Literature (CINAHL) and the metasearch engine Exploraevidencia. Websites of palliative care organisations (Sociedad Española de Oncología Médica [SEOM], Sociedad Española de Cuidados Paliativos [SECPAL], European Association for Palliative Care [EAPC], International Association for Hospice and Palliative Care [IAHPC], National Hospice and Palliative Care Organisation [NHPCO], Registered Nurses’ Association of Ontario [RNAO] and Edmonton Zone Palliative Care Programme, located in the SEOM Manual of Care [19] were also reviewed.

Searches used both standardised terms Medical Subject Headings (MeSH), CINAHL Subject Headings (palliative care, hospice and palliative care nursing, hospice care, palliative medicine, practice guidelines, patient care, hospice patients, terminal care) and free-language terms (end of life, last days of life). The publication time frame was set from January 2016 to December 2025 to obtain the most updated recommendations. Searches were executed in December 2025.

CPGs published in English or Spanish were accepted. Guidelines published in local languages with local reach were not taken into account. After reviewing electronic databases, studies were selected by title in each database. Duplicates were removed and abstracts were reviewed by two researchers independently to include only CPGs. Discrepancies were resolved after discussion and consensus between the two researchers. Records were imported into Rayyan systematic review software [20] for deduplication and title/abstract screening. Full-text references were managed using Mendeley Academic Reference Manager. Resulting documents were reviewed in full text, verified to be CPGs and, if not, discarded. In CPGs, objectives and clinical questions answered in each were reviewed. In case of several publications of the same guideline, the guideline corresponding to the latest revision by the guideline development group was selected. In case of discrepancy, an external expert independently re-evaluated the guide.

The methodological quality of these documents was assessed using the Appraisal of Guidelines for Research Evaluation Instrument II tool (AGREE II) (AGREE Next Steps Consortium, 2009). CPGs were assessed individually by four evaluators. The domain scores were calculated by summing all the points of the individual items of the domain scored by experts and standardising the total as a percentage by subtracting the minimum possible score from the obtained score and dividing the result by the maximum possible score minus the minimum possible score. The selection of guidelines for inclusion was not based solely on the overall score but on performance across individual domains, with particular weight given to Domain 3 (Rigour of Development) and Domain 6 (Editorial Independence), considered critical for assessing the trustworthiness of guideline recommendations, and Domain 5 (Applicability). Inter-rater discrepancies were resolved by consensus among the four reviewers. A comparative table of domain scores for all evaluated CPGs was drawn up (Table 2).

Relevant data were extracted from each included guideline using a standardised extraction form, covering guideline title, issuing organisation, country, year of publication, target population, clinical scope, recommendations addressing the last days of life, strength of recommendation, and evidence grading system used.

Given the heterogeneity of the included guidelines in terms of structure, terminology, and recommendation grading systems, a narrative synthesis approach was adopted. Recommendations from the eight guidelines included were categorised into 12 decision areas that were generated inductively during the review process: identification of end-of-life situation; end-of-life care communication and decision-making with patients and caregivers; access to multidisciplinary care; hydration and nutrition; symptom management: general approach; symptom management: dyspnoea and secretions; pain; nausea and vomiting; anxiety, delirium and distress; rattles; grief and bereavement; and invasive treatments (Table 3).

Ethical review and approval were not required for this study because no human participants, patients, or sensitive data were involved.

## 3. Results

### 3.1. Search Results

**Figure 1:**
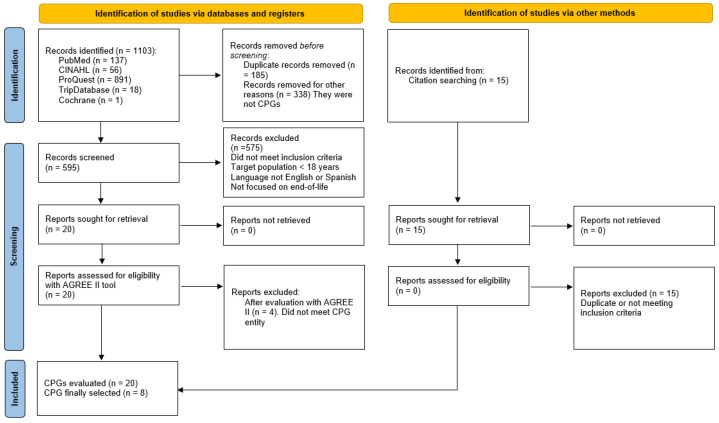
Flow chart of study section. Adapted from PRISMA [17].

A total of 1103 records were identified through database searching (PubMed n = 137; CINAHL n = 56; ProQuest n = 891; TripDatabase n = 18; Cochrane n = 1), and an additional 15 records were identified through citation searching, yielding 1118 records in total. Following removal of 185 duplicate records and 338 records that did not meet the CPG definition, 595 records were screened by title and abstract. Of these, 575 were excluded for not meeting the inclusion criteria (paediatric population, language other than English or Spanish, or not focused on the last days of life), leaving 20 reports for full-text retrieval. All 20 were successfully retrieved. Four were subsequently excluded as they did not meet the criteria of a CPG entity [21,22,23,24], leaving 16 guidelines for quality appraisal with the AGREE II tool. Following quality appraisal, eight guidelines were excluded and eight were finally selected. The screening process is described in Figure 1.

### 3.2. Characteristics of Included Guidelines

Following the quality appraisal, 8 of the 16 CPGs were removed and eight were finally selected. The eight included guidelines were published between 2018 and 2021, originating from five regions: the United Kingdom (NICE 2020 [25], NICE 2019 [26]), Spain (GPCAPASUD [27]), the United States (ICSI 2020 [28], NCHPC 2018 [29]), Canada (RNAO 2020 [30]), and Europe (ESMO 2021 [31], ESMO 2018 [32]). All guidelines addressed adult patients in the last days of life, either exclusively or as an identifiable component of broader end-of-life care guidance. From a methodological point of view, three CPGs—NICE 2019 [26], NICE 2020 [25], and ICSI [28]—used the GRADE system [33]; two others combined GRADE with additional tools such as GRADE-CERQual [34] (RNAO 2020); and two used their own internal grading system. Full details of the context, objectives, methodology, and recommendations of each guideline are provided in Supplementary Table S1. The details of the AGREE II scores are described in Table 2.

### 3.3. AGREE II Assessment

The methodological quality scores obtained with AGREE II for all 16 evaluated CPGs are shown in Table 2. Overall scores ranged from 25% (ACPGBI [35]) to 100% (NICE 2020 [25]).

The two highest-scoring guidelines were NICE 2020 [25] (overall: 100%; D3: 97%; D6: 94%) and GPCAPASUD [27] (overall: 96%; D3: 91%; D6: 87%), followed by NICE 2019 [26] (overall: 92%; D3: 95%; D6: 94%), ICSI 2020 [28] (overall: 92%; D3: 91%; D6: 64%), NCHPC 2018 [29] (overall: 92%; D3: 86%; D6: 96%), RNAO 2020 [30] (overall: 87%; D3: 76%; D6: 62%), and both ESMO 2021 [31] and ESMO 2018 [32] (overall scores: 87% and 71% respectively; D3: 72% for both).

Among the excluded guidelines, BCCPC 2019 [36] presented a particularly instructive case: despite an overall score of 75%, it was excluded due to critically low scores across three key domains—Domain 3 (62%), Domain 5 (49%), and Domain 6 (12%)—indicating insufficient methodological rigour, limited practical applicability, and a lack of transparency regarding conflicts of interest. The remaining excluded guidelines obtained overall scores below 65%, with several scoring critically low in Domain 3 (ACPGBI [35]: 11%; PSCHNC [37]: 15%) or Domain 6 (HISSPCG [38] and PSCHNC [37]: 0%).

Across all included guidelines, the domain with the highest scores was Clarity of Presentation (Domain 4), followed by Scope and Purpose (Domain 1). Conversely, the lowest scores were consistently observed in Domain 5 (Applicability), where even among the selected guidelines scores ranged from 49% (BCCPC, excluded) to 100% (NICE 2020 [25]), and three excluded guidelines scored below 30%.

From a methodological point of view, three of the selected CPGs—NICE 2019 [26], NICE 2020 [25] and ICSI [28]—opted for the GRADE system [33], while two others used other tools in addition to the GRADE method, such as AGREE II: RNAO 2020 and GRADE CerQual [34]. Finally, two of them used their own system [27,29].

### 3.4. Coverage of Decision Areas

Based on the 10 principles recommended by the International Collaborative for Best Care for the Dying Person, a group of experts identified twelve groups of topics into which the recommendations contained in eight guidelines selected were grouped. To present the strength of recommendations a mixed method was used: the one used by the GRADE working group [33] combined with additional information. For more information (context, objectives, methodology and recommendations of every guideline), see Supplemental File Table S1, “Recommendation in selected Guidelines”

CPGs contained specific recommendations for healthcare providers in caring for patients nearing the end of life. These include communication strategies, symptom management (pain, dyspnoea, nausea, etc.), decision-making processes, and considerations for artificial hydration and nutrition. An interdisciplinary team approach was included involving physicians, nurses, social workers, and other healthcare professionals. Moreover, there were recommendations on the importance of involving both patients and caregivers in the care process, including shared decision-making and providing comprehensive support. There were also recommendations on the need to respect patient and family beliefs, values, and cultural backgrounds as well as social, psychological issues, grief and bereavement in palliative care settings.

From the twelve areas of decision identified, recommendations on end-of-life care communication and decision-making with patients and caregivers and access to multidisciplinary care were present in most of the CPGs. The decision areas with fewer recommendations present in CPGs were management of rattles, and decision on invasive treatments. Symptom control is present in many CPGs, with strong recommendations in most cases (Table 3).

### 3.5. Strength of Recommendation and Summary Gaps

Regarding the category referring to the recognition of a last days of life situation, all guidelines provide recommendations, except four [25,29,30,32]. The strength of recommendation was strong across all guidelines, except in NCHPC [29] and GPCAPASUD [27], which have a strong and weak level of recommendation regarding that item.

On the other hand, regarding the communication and information category, five guidelines provide recommendations, four of which had a strong level of recommendation: NICE 2019 [26], NICE 2020 [25], ESMO 2021 [31], GPCAPASUD [27], and NCHPC [29] presented a mix of strong and weak levels of recommendation for every item.

In the area of ethical and legal aspects of care, life-sustaining treatments, shared decision-making and person-centred care, four CPGs strongly recommend these: RNAO [30], NICE 2019 [26], NICE 2020 [25] and GPCAPASUD [27].

Regarding the social and psychological issues group only half of the selected guidelines—NCHPC [29], ICSI [28], NICE 2020 [25], and ESMO 2021 [31]—make recommendations in this regard, and these are mostly a strong level of recommendation.

No contradictions in recommendations were found between guidelines, although there are differences in the strength of the recommendation (strong or weak). NCHPC [29] is the guideline with the weakest recommendations or with a higher mix of strong and weak levels of recommendation for every item.

## 4. Discussion

This review aimed to identify and evaluate contemporary CPGs in the field of end-of-life care for adult patients in the LDS, their families, and caregivers for the care of patients at the end of life and their families.

Recognition of the end of life obtained strong recommendations in most of the CPGs, which is consistent with the importance of acknowledging this scenario. Nevertheless, despite the importance to prioritize and select interventions, health professionals continue to carry out the continuation of numerous interventions and therapeutic decisions that are typical for acute disease and far from the objectives of palliative care [39].

Effective communication is essential to identify the needs of patients and helps in the decision-making of patients and their families, essential at the end of patients’ lives. Patients and their relatives want healthcare professionals to pay attention to the patient’s disease and symptoms, to who the patient is beyond the illness, and to the role of the relative [40]. Most of the CPGs addressed this area of decision, although evidence suggests that professional competence in this area still needs improvement [41,42,43]. Knowledge transfer and implementation in this area should therefore incorporate training in communication skills, as without such interventions, the impact of CPG recommendations is likely to remain limited.

Few CPGs include recommendations for psychological needs assessment and support, despite it being a key ingredient of a good quality of end-of-life care [44]. Healthcare professionals caring for patients at the end of life should be encouraged to increase psychological support to patients to improve meaning or spiritual well-being [45] and future CPGs should extend their scope to include recommendations for psychological assessment, support and referral.

Only three CPGs included recommendations on referral for cultural, religious and spiritual assessment, NCHPC [29], RNAO [30] and Care of the adult cancer patient at the end of life: ESMO clinical practice guidelines [31]. This represents a notable gap, given the growing body of evidence on the clinical relevance of spirituality in oncology and supportive care. Recent systematic evidence demonstrates that greater spiritual well-being is consistently associated with improved quality of life, reduced psychological distress, and better coping across multiple cancer types [45]. Furthermore, spiritual well-being has been shown to moderate anxiety and depression in cancer patients, underscoring its role not only as a personal resource but as a clinically meaningful outcome in its own right [46,47]. It has been recommended to incorporate spiritual assessment into the care of patients with serious illnesses, as Balboni et al. identify it as a key component of person-centred care [48]. At the same time, there is a lack of guidance regarding end-of-life decision-making among ethnocultural minorities, despite evidence showing that culturally adapted decision-making tools improve outcomes for patients and their families [49,50]. Recent studies confirm that culture strongly influences how death is understood, which practices are accepted or rejected, and how decisions are made during the dying process. Factors such as religion, ethnicity, and family values significantly shape attitudes toward palliative care, advance care planning, and end-of-life rituals [51]. Moreover, cultural representations of death influence not only the meaning of loss, but also experiences of grief and preparation for dying [52], highlighting the need for person-centred care that addresses physical, spiritual, symbolic, and cultural dimensions of dying [53]. This is particularly relevant in multicultural settings, where values related to spirituality, family, and community play a central role in end-of-life experiences [54].

The low scores in the Applicability domain of the AGREE II instrument highlight important limitations of the included guidelines, particularly regarding implementation barriers and facilitators, practical application strategies, resource implications, and monitoring criteria. These findings have implications for guideline developers, clinicians, managers, and implementation science. Future guidelines should include practical implementation tools, such as decision aids, care pathways, and educational resources, while also addressing organisational and cultural barriers, resource needs, and evaluation indicators. For clinicians and healthcare managers, the limited applicability guidance may hinder the integration of recommendations into routine care, especially in multicultural end-of-life settings, making local protocols and staff training necessary. From an implementation science perspective, greater attention should be paid not only to methodological quality, but also to the feasibility, acceptability, sustainability, and real-world impact of guidelines.

The most important limitation is that, due to the lack of clinical practice guidelines dealing exclusively with the care of patients in the last days of life, the results obtained and analysed have also been from some guidelines dealing with the end of life in a general way. Another important limitation has been the difficulty in analysing the strength of the recommendations, as in some of the selected guidelines this is not clearly expressed [29]. Furthermore, the results of this review cannot be extended to CPGs for the LDS in paediatric patients or those admitted to the ICU. Additionally, other important limitations include the potential influence of language restrictions and the possible omission of the relevant grey literature, which may have limited the identification of all available evidence. We should also consider the variability in the definitions of the last days of life across studies and guidelines, as well as the heterogeneity in guideline development systems, methodological approaches, and healthcare contexts, all of which may affect the comparability and consistency of recommendations. Finally, the potential subjectivity involved in categorizing and synthesizing recommendations should be recognised, despite efforts to ensure methodological rigour and reviewer agreement throughout the study.

All the selected guidelines considered the opinion of the relevant groups of patients and relatives, which is essential in the field of end-of-life care.

## 5. Conclusions

The present systematic review found agreement across last-days-of-life clinical practice guidelines with mostly strong recommendations for recognition of the last days of life, communication and information, ethical and legal aspects of care, and interdisciplinary teams and coordination. Recommendations on symptom control and the care of patients were mixed—weak and strong. It should also be noted that the recommendations are limited by the heterogeneity and methodological variability of the included guidelines. Future clinical practice guidelines should better address social, cultural and spiritual issues.

Our findings may be useful for professionals caring for patients at the end of life and health system managers to identify guidelines and recommendations identified as key to their care. The highlighted gaps in patient and caregiver perspectives should serve as a basis for the development of future guidelines.

## Figures and Tables

**Table 1:** PICO framework. LDS: last days of life situation.

Population	Adult patient in the last days of life and their relatives and carers
Intervention	Recommendations for action and care in the LDS with a high degree of evidence collected in quality clinical practice guidelines
Comparison	Not applicable
Outcomes	Physical, mental and spiritual well-being of patients and caregivers

**Table 2:** Guidelines included and excluded (D = domain).

		D.1	D.2	D.3	D.4	D.5	D.6	Overall Score	Recommendations
**Included**	National Institute for Care and Health Excellence (NICE). United Kingdom. 2020	100	100	97	100	100	94	100	YES
	Guia de Practica Clinica sobre atencion paliativa al adulto en situacion de ultimos dias (GPCAPASUD). Spain. 2021	96	96	91	94	83	87	96	YES
	National Institute for Care and Health Excellence (NICE). United Kingdom. 2019	97	99	95	97	83	94	92	YES
	Institute for Clinical Systems Improvement (ICSI). United States. 2020	99	69	91	90	87	64	92	YES
	National Coalition for Hospice and Palliative Care (NCHPC). United States. 2018	96	92	86	92	87	96	92	YES
	Registered Nurses’ Association of Ontario (RNAO). Canada. 2020	93	92	76	86	75	62	87	YES
	European Society for Medical Oncology (ESMO). 2021	78	62	72	94	61	79	87	YES
	European Society for Medical Oncology (ESMO). 2018	79	46	72	97	36	64	71	YES
**Excluded**	B.C. Centre for Palliative Care (BCCPC). Canada. 2019	96	97	62	93	49	12	75	YES
	BC Guidelines (BCG). Canada. 2017	93	57	34	82	42	37	62	YES with MOD.
	Japanese Society of Palliative Medicine (JSPM). Japan. 2019	78	92	51	86	32	46	62	YES
	Sociedad Española y Portuguesa de Medicina Interna (SEPMI). Spain. 2020	76	62	36	75	30	64	58	YES with MOD.
	Healthcare Improvement Scotland. Scottish Palliative Care Guidelines—Care in the Last Days of Life (HISSPCG). United Kingdom. 2019	80	30	26	74	13	0	37	YES with MOD.
	Palliative and supportive care in head and neck cancer: United Kingdom National Multidisciplinary Guidelines (PSCHNC). United Kingdom. 2016	82	61	15	92	29	0	33	YES with MOD.
	Association of Coloproctology of Great Britain & Ireland (ACPGBI). 2017	72	68	11	85	30	33	25	NO
	National Institute for Care and Health Excellence (NICE). United Kingdom. 2016	94	96	60	86	55	81	62	YES

D.1: Scope and Purpose; D.2: Stakeholder Involvement; D.3: Rigour of Development; D.4: Clarity of Presentation; D.5: Applicability; D.6: Editorial Independence. AGREE II: Appraisal of Guidelines for Research and Evaluation instrument, version II. CPG: clinical practice guideline. NICE: National Institute for Care and Health Excellence; GPCAPASUD: Guía de Práctica Clínica sobre Atención Paliativa al Adulto en Situación de Últimos Días; ICSI: Institute for Clinical Systems Improvement; NCHPC: National Coalition for Hospice and Palliative Care; RNAO: Registered Nurses’ Association of Ontario; ESMO: European Society for Medical Oncology; BCCPC: B.C. Centre for Palliative Care; BCG: BC Guidelines; JSPM: Japanese Society of Palliative Medicine; SEPMI: Sociedad Española y Portuguesa de Medicina Interna; HISSPCG: Healthcare Improvement Scotland Scottish Palliative Care Guidelines; PSCHNC: Palliative and Supportive Care in Head and Neck Cancer; ACPGBI: Association of Coloproctology of Great Britain & Ireland.

**Table 3:** Decision areas covered by the selected guidelines.

	NICE 2020	GPCAPASUD 2021	NICE 2019	ICSI 2020	NCHPC 2018	RNAO 2020	ESMO 2018	ESMO 2021
1. Identification of end-of-life situation		X	X	X				X
2. End-of-life care communication and decision-making with patients and caregivers	X	X	X	X	X	X		X
3. Access to multidisciplinary care	X		X	X	X	X		X
4. Hydration and nutrition		X						X
5. Symptom management: general approach		X						
6. Symptom management: dyspnoea and secretions		X						X
7. Symptom management: pain		X					X	X
8. Symptom management: nausea and vomiting		X						X
9. Symptom management: anxiety, delirium and distress		X					X	X
10. Symptom management: rattles		X						
11. Grief and bereavement				X	X			
12. Invasive treatments								X

X: recommendation present. NICE: National Institute for Care and Health Excellence; GPCAPASUD: Guía de Práctica Clínica sobre Atención Paliativa al Adulto en Situación de Últimos Días; ICSI: Institute for Clinical Systems Improvement; NCHPC: National Coalition for Hospice and Palliative Care; RNAO: Registered Nurses’ Association of Ontario; ESMO: European Society for Medical Oncology.

## Data Availability

The original contributions presented in this study are included in the article/Supplementary Material. Further inquiries can be directed to the corresponding author.

## Supplementary Materials

The following supporting information can be downloaded at: https://www.mdpi.com/article/10.3390/jcm15124407/s1, Table S1: Recommendation in selected Guidelines.

## Abbreviations

The following abbreviations are used in this manuscript:

LDS	Last days of Life Situation
ICBCDP	International Collaborative for Best Care for the Dying Person
EOLRG	End-of-Life Research Group
CPGs	Clinical practice guidelines
PICO	Population, Intervention, Comparison, and Outcome
PRISMA	Preferred Reporting Items for Systematic Review and Meta-Analyses
ICU	Intensive Care Unit
CINAHL	Cumulative Index of Nursing and Allied Health Literature
SEOM	Sociedad Española de Oncología Médica
SECPAL	Sociedad Española de Cuidados Paliativos
EAPC	European Association for Palliative Care
IAHPC	International Association for Hospice and Palliative Care
NHPCO	National Hospice and Palliative Care Organization
RNAO	Registered Nurses’ Association of Ontario
MeSH	Medical Subject Headings
AGREE	Appraisal of Guidelines for Research Evaluation Instrument
NCHPC	National Coalition for Hospice and Palliative Care
NICE	National Institute for Care and Health Excellence
ICSI	Institute for Clinical System Improvement
ACPGBI	Association of Coloproctology of Great Britain & Ireland
HISSPCG	Healthcare Improvement Scotland. Scottish Palliative Care Guidelines
PSCHNC	Palliative and supportive care in head and neck cancer
ESMO	European Society for Medical Oncology
GPCAPASUD	Guía de Práctica Clínica sobre Atención Paliativa al Adulto en Situación de Últimos Días
BCG	BC Guidelines
JSPM	Japanese Society of Palliative Medicine
SEPMI	Sociedad Española y Portuguesa de Medicina Interna
BCCPC	B.C. Centre for Palliative Care
